# Irregularities and How to Correct Some of Them

**Published:** 1902-11-15

**Authors:** J. H. Stofflett


### IRREGULARITIES AND HOW TO CORRECT SOME OF THEM.

BY J. H. STOFFLETT, D.D.S.

Read before the Southwestern Michigan Dental Society, September 9, 1902.

The teeth of the human family, when in their normal position, are arranged in symmetrical order, their outline describing very nearly a semi ellipse; their variation being a slight flattening in the incisor region and a tendency toward angularity in the region of the cuspids.

Any marked deviation from this constitutes what is known to the dental profession as an irregularity.

Dental irregularities may be divided into two general varieties, one where certain teeth stand out of line or are improperly placed in line, the other where the teeth all stand in line but where their outline is so changed from the normal as to constitute a deformity or malformation.

Either of these, or what often occurs, a combination of the two, is what calls for the interferance of the dentist.

Irregularities may be either hereditary or acquired. The former resulting from causes operating before the birth of the individual or the eruption of the teeth and the other from circumstances attending the eruption or following it. Certain families manifest dental peculiarities which distinguish them from generation to generation showing that there is a strong tendency on the part of nature to reproduce herself.

The offspring resembling its parent in its dental makeup just the same as it does in form, feature or movement; or it may inherit some of the peculiarities from one parent and some from the other, thus we have a blending of the peculiarities of the parents in the offspring. This being the case, the one parent may have a small jaw while the other has large teeth. The child may inherit the small jaw of the one and the large teeth of the other, thereby causing a crowded or malplaced condition of the teeth in insufficient room to accomodate them. This theory is generally accepted as the cause of irregularities where the intermingling of races is common. Foetal inervation is another supposed cause.

The acquired form of irregularity is known to be partly due to the premature extraction or the long retention of the deciduous teeth ; to accidents, to thumb or lip sucking, tardy eruption of certain teeth, mouth breath the theory of Dr. Kingsley associating the inervationing, and of the fifth nerve brought on by the methods of life in vogue to-day among the higher classes of society. Whatever their origin, the evils attending are many and great and this should be kept in mind by the dentist and patient.

The most prominent ones are distortion of the features interference with speech, predisposition to decay, indigestion and injury to health due to defective mastication. The last named evil should be the greatest incentive to the undertaking of an operation for the correction of irregularity. The deviation from a normal standard, both in regard to the shape of the arch and the arrangement of the teeth, are greater to-day than they were in the early days of dentistry.

It is quite a common occurence to see an irregularity of one or more of the six upper anterior teeth, and here the dentist is called upon to correct or improve nature's imperfections. To-day, with the better knowledge of the subject and improved appliances, results can be obtained that were, in the pioneer days of the profession, thought impossible. There is a strong tendency on the part of nature to correct itself, but to do this, she must have time and occasionally a little mechanical assistance. Before undertaking to correct an irregularity, several conditions should be thoroughly weighed, namely ; general and local health, age of patient, sex and nature of alveolar process. A person of extreme nervous temperament is scarcely a fit subject for the endurance of the pain and inconvenience neccessary for the correction of irregular teeth, because of nervous excitability aroused, which is liable to impair health which is of more importance than the local good.

The best time for the correction of irregularities is between the years of twelve and twenty-one, but some changes may be made as late as twenty-eight or thirty. The age of fourteen is a critical period in the life of a female. At this age she is quite irritable and anything that would increase this irritability may be the means of impairing health for a life time. In any case when any marked changes are to be brought about in a female of this age, I consider it good judgment to postpone such changes until some later time. The boy having no such special period, his case is only considered from a standpoint of health. The summer vacation is usually the best time for such operations on children, because at this time they are under less mental strain. In youth, the alveolar process is of loose structure and is more capable of responding to mechanical impressions then in one of dense structure. The process of absorption and the foundation of new tissues in the relieved parts is more rapid in young people than those of mature age. Having duly considered the conditions and having decided to assist nature in bringing about the desired changes, the question arises, by what mechanical means are we to bring about this correction.

This of course will depend upon the changes to be made. No one appliance will meet all of the requirements in an individual case where various changes are to be made. Each case requires more or less inventive ingenuity on the part of the operator. Taking a case where there is a protrusion of one or all of the upper anterior teeth, I have obtained the best results with an appliance made of vulcanite rubber, covering the molar and bicuspid teeth, and extending up over the buccal side a short distance on the gum, attaching to this a piece of No. 15 B & S gauge German silver wire and extending this around in front of the anterior teeth so as to be quite tight and fastening it to the vulcanite piece on the opposite side, then by daily driving orange wood wedges between the teeth and wire, the desired changes are made in a short time. With this appliance I have a firm foundation anchorage and I can apply any required amount of force necessary and in whatever direction it is needed. In a case where one or more of the teeth are inside of the arch, I make a plate covering the roof of the mouth and the posterior teeth; the plate extending tight against the anterior teeth and covering about two-thirds of lingual side of them, then by driving wedges between the teeth and the plate, I bring them into line.

If the case requires the drawing back of some teeth and the crowding out of others, I make use of the last named plate with the wire attachment and apply the wedges in opposite directions.

If it is necessary to rotate a tooth in its socket, say for instance a central incisor, I apply two wedges, one on the labial side and one on the lingual side and by renewing these daily the desired change will soon be brought about.

### DISCUSSION.

Dr. Ricks:—I have a case for which I made an appliance last week. The two lateral incisors are erupted outside and above the arch, and very short. I fitted bands to each tooth with a small tube soldered parallel to the occlusal plane. I then banded the second deciduous molars with similar band and tube. A wire threaded on one end for a nut was passed through these tubes from the molar of one side to the molar on the other. The end opposite the threaded one was fastened to its tube, and a nut placed on the screw end, enables me to tighten the wire a little each day. The result is accomplishing the work satisfactorily.

Dr. Taft:—Care should be exercised in using the deciduous molars as anchorages. The roots are being absorbed by the permanent molars. Intermittent pressure, with its periods of rest, is a preferable method of applying force to the constant pressure of an elastic spring. It is not liable to cause excessive absorption of the anchorage teeth. Sometimes the constant pressure of an elastic spring or rubber band is indicated and may be the better treatment. A knowledge of the object sought will determine the best method.

Dr. Runyan:—I have experienced difficulty in making rubber plates to cover the molar and bicuspid teeth that would fit securely enough to permit using them as bases from which to exert much force. I would like to ask the essayist how he manages this. I have never been able to use these plates satisfactorily.

Dr. Stofflett:—I take accurate impressions of the teeth and by carving the plaster models at the buccal cervical margins, the rubber being elastic will spring over the teeth and grip them firmly, the lower teeth will also help to retain the plate by the force of occlusion. After fitting the wax base plate to the plaster model, remove it and place it in the mouth and ask the patient to close the teeth ; this will give the prints of the cusps of the lower teeth and when the plate is vulcanized and put in place will materially help to retain it in position.

DR. ROE :—I have had personal experience with plates made as the essayist suggests and can vouch for their retention abilities.

DR. STOFFLETT :—At first I was doubtful of being able to accomplish much with this plan of regulating. But successful results of the past year in a number of cases, some of which I show by the models exhibited here, seem to justify the procedure. In one case I moved for a lady, twenty-six years of age, the right lateral incisor from a lingual position, where it was crowded behind the cuspid and central, to its proper place in the arch in twenty days, simply by means of the wedges forced up between the plate and the tooth. And this too after I had failed to accomplish the object by means of a jack screw set in a plate ; the operation of which resulted only in elongating the lateral without driving it forward at all.

DR. ROE :—I have a case which I wish to report and would like some advice as to its treatment. A boy thirteen years of age has in position the anterior permanent incisors and cuspids and the first molars in each jaw, but none of the bicuspids are erupted. The lower incisors occlude outside of the upper, and the lower cuspids are not fully erupted. The jaw is narrow and contracted. The first molars are decayed, but I filled them temporarily. There are at this time no indications of the bicuspids.

DR. WATTING :—It is difficult to indicate treatment for such a case without seeing it, and thus securing more information as to the exact conditions. It is possible or probable that the bicuspid may never erupt, it may be necessary to supply their places with bridges. The mal-occlusion of the incisors should be corrected at any rate, this will serve to close the spaces in the bicuspid regions. If the first molars are utilized for bridges the pulps should be removed, and this necessarily shortens their life, and the result will be that the space will be increased. A band on the cuspids will also threaten their lives and usefulness, so that it becomes rather hazardous to undertake bridges in such a case. Personally, I am not an advocate of extensive pieces of bridge work.

